# Proteomic Analysis of Serum Lysine Acetylation in Uyghur Patients With T2DM

**DOI:** 10.3389/fmolb.2022.787885

**Published:** 2022-03-30

**Authors:** Liang Yin, Gang Feng, Chun Huang, Weijuan Cai

**Affiliations:** ^1^ Department of Endocrinology and Metabolism, Zhanjiang Central Hospital, Guangdong Medical University, Zhanjiang, China; ^2^ Department of Endocrinology and Metabolism, Affiliated Hospital of Qingdao Binhai University, Qingdao, China; ^3^ Clinical Research Institute, Affiliated Zhanjiang Central People’s Hospital of Guangdong Medical University, Zhanjiang, China

**Keywords:** lysine acetylation, proteomics, T2DM, histones, Uyghurs

## Abstract

Lysine acetylation is a reversible modification process after protein translation, which plays a key regulatory role in various metabolic diseases such as diabetes. The prevalence of type 2 diabetes mellitus (T2DM) in the Uyghur population is high, but the acetylation status of proteomics in Uyghur with T2DM is still unclear. Herein, we performed a quantitative proteomic study of lysine acetylation in T2DM patients using Tandem Mass Tags (TMTs) labeling, acetylation enrichment techniques, and high-resolution liquid chromatography-tandem mass spectrometry. We quantified 422 acetylation sites on 120 proteins, of which 347 sites of 103 proteins contained quantitative information. Compared with the control, we found that a total of eight acetylated sites within proteins were significantly differentially expressed with three upregulated and five downregulated, including histones H4 and H3.3C. Meanwhile, we completed bioinformatics analysis, including protein annotation, functional classification, functional enrichment, and cluster analysis, based on functional enrichment. In addition, the mRNA (ApoB-100, histones H4 and H3.3C) and protein (histones H4 and H3.3C) levels were verified through 60 samples. Besides, we also performed histone H4 chromatin immunoprecipitation analysis at the level of INS-1 cells. These could be potentially useful markers for the prediction of prediabetes and also provided a basis for the pathogenesis of T2DM.

## Introduction

Type 2 diabetes mellitus (T2DM) is a disease in which genetic and environmental factors work together. It has become one of the major chronic diseases that pose a serious threat to human health. At present, China has become the country with the largest number of T2DM patients (Ma and Chan, 2013). The prevalence of rapid growth is related to risk factors such as eating habits, living environment, and life stress. The burden of disease caused by T2DM is very serious, and T2DM control in China cannot be delayed. However, the exact molecular mechanism of the disease is still unclear.

Studies have found that the occurrence and development of diabetes are affected by genetic and epigenetic factors (Rosen et al., 2018). Epigenetics was originally a concept proposed by Waddington (1939). Epigenetics currently refers to altering gene expression levels by genetic modification without altering the nucleotide sequence, resulting in a heritable phenotype. Lysine acetylation modification is the process of adding an acetyl group to a lysine residue of a protein by the action of an acetyltransferase. Protein acetylation is mainly regulated by acetylase and deacetylase and is involved in many biological processes such as transcription, metabolism, cell signal transduction, and apoptosis (Hebert et al., 2013; Ahmed et al., 2017). It is an important reversible protein post-translational modification process in cells. In addition, histone modification referred to the acetylation and deacetylation, methylation and demethylation, ubiquitination, and deubiquitylation of the N-terminal amino acid residues of chromatin histones (Jenuwein and Allis, 2001; Berger, 2007). Histone acetylation is one of the important mechanisms in epigenetics. Although an increasing number of reports described the association of distinct histone modifications with autoimmunity (Villeneuve et al., 2008) and carcinogenesis (Wilson et al., 2009), little is known about its role in diabetes.

Xinjiang is a multi-ethnic gathering area. Many diseases have their national particularities. Uyghurs are the main ethnic group in Xinjiang. The population of Xinjiang Uyghur has obvious risk factors, such as overweight and insulin resistance, and the prevalence of diabetes was much higher than for other ethnic minorities (Tao et al., 2008). The reasons for this phenomenon are still unknown. The gradually increasing data displayed that epigenetic mechanisms were involved in the development of T2DM (Pinney and Simmons, 2010). However, the regulation mechanism of acetylated proteomics in Uyghur patients with T2DM is still unclear. Herein, this project combines a series of cutting-edge technologies such as TMT labeling, high-performance liquid chromatography grading technology, acetylation peptide enrichment technology, and mass spectrometry-based quantitative proteomics technology to quantitatively analyze acetylation in samples. The aim is to provide a strong basis for the in-depth study of protein acetylation in Uyghur patients with T2DM.

## Materials and Methods

### Sample Collection

Patients group (P): we collected three blood samples (age 67 ± 8.64 years) in Uyghur patients with T2DM. T2DM diagnosis meets the 1999 World Health Organization Diabetes Diagnostic Criteria. According to medical history and family history, type 1 diabetes, young-onset adult diabetes, and mitochondrial diabetes are excluded. The patients have lived in Xinjiang for a long time, and three generations have not been married to other races, with no autoimmune disease; no serious heart, liver, and kidney disease; no malignant tumors; and non-pregnant or lactating women. Normal control group (N): three blood samples of normal glucose tolerance (NGT) (age 68.3 ± 6.94 years) were taken from Uyghur populations, respectively. All selected subjects were measured for blood pressure, body weight, height, waist circumference, hip circumference, fasting blood glucose (FPG), 2 h plasma glucose (2hPG), fasting insulin (FINS), fasting C-peptide (FCP), glycated hemoglobin (HbA1c), and serum lipids, among others. The insulin resistance index of the steady-state model was calculated (HOMA-IR = FPG×FINS/22.5). Characteristics of sample collection of proteomic analysis are shown in Supplementary Table S1. In addition, another 60 Uyghur patients with T2DM and 60 sex- and age- (±3 years) match healthy controls were recruited from the same hospital used for further validation. The study was approved by the ethics committee of the First Affiliated Hospital of Shihezi University School of Medicine, and the consent was obtained from all patients. About 5 ml of fasting venous blood was collected from each subject (EDTA anticoagulation) and centrifuged at 3,000 rpm for 10 min. Separated serum was stored at −80°C.

### Protein Extraction of Serum

Samples were taken from the −80°C freezer, centrifuged at 12,000 g for 10 min at 4°C, and the supernatant of the cell debris was removed and transferred to a new centrifuge tube. Then, the top 12 high abundance proteins were removed by Pierce™ Top 12 Abundant Protein Depletion Spin Columns Kit (Thermo Fisher, United States). After 7-8-fold dilution, the protein concentration was determined using the BCA kit (TIANGEN, China) according to the manufacturer’s instructions.

### Trypsin Digestion

Take the same amount of protein in each sample, add the same volume of pre-chilled acetone, vortex, and mix. Then, add four times the volume of the pre-chilled acetone and precipitate at −20°C for 2 h. Centrifuge at 4,500 g for 5 min, discard the supernatant, and wash the precipitate two to three times with pre-cooled acetone. After drying the precipitate, add an appropriate amount of 8 M urea, reconstitute it with ultrasound, adjust the volume to the same with the lysate, then add dithiothreitol to make the final concentration of 5 mM, and reduce at 56°C for 30 min. After that, iodoacetamide was added to make the final concentration of 11 mM and incubated at room temperature in the dark for 15 min. Then, the protein sample was diluted by adding 100 mM TEAB to achieve a urea concentration of less than 2 M. Trypsin (Promega, United States) was added at a mass ratio of 1:50 (trypsin: protein) and digested overnight at 37°C. Add trypsin at a mass ratio of 1:100 (trypsin: protein) and a second 4 h digestion.

### Tandem Mass Tags

The trypsin-digested peptides were desalted using Strata X C18 (Phenomenex, United States) and vacuum freeze-dried. Dissolve the peptides with 0.5 M TEAB and label the peptides according to the manufacturer’s protocol for Tandem Mass Tags (TMTs) kit (Thermo Fisher, United States). The simple operation was as follows: one unit of TMT reagent was thawed, dissolved in acetonitrile, mixed with the peptide, and incubated at room temperature for 2 h. Next, the labeled peptides were mixed, desalted, and dried by vacuum centrifugation.

### High-Performance Liquid Chromatography Fractionation

The tryptic peptides were fractionated by high pH reverse-phase HPLC using Thermo Betasil C18 column (5 μm particles, 10 mm ID, 250 mm length). Briefly, the peptides were separated into 60 fractions using a gradient of 8–32% acetonitrile (pH = 9.0) over 60 min. Then, the peptides were combined into four fractions and vacuum freeze-dried for subsequent operations.

### Affinity Enrichment

To enrich modified peptides, tryptic peptides were dissolved in IP buffer solution (100 mM NaCl, 1 mM EDTA, 50 mM Tris-HCl, 0.5% NP-40, pH of 8.0). Firstly, the supernatant was transferred into the anti-acetyllysine antibody-conjugated agarose beads (PTM-104, PTM Biolabs, Hangzhou, China), placed on a rotary shaker at 4°C, and incubated overnight. Secondly, the beads were washed four times with IP buffer solution and twice with deionized water. The bound peptides were eluted using a 0.1% trifluoroacetic acid eluate and eluted three times. The eluate was collected and vacuum-dried, finally desalting according to C18 ZipTips (Millipore, United States) instructions, vacuum drying, and refrigerating for LC-MS/MS analysis.

### Liquid Chromatography-Tandem Mass Spectrometry Analysis

Solvent A is a solution containing 0.1% formic acid and 2% acetonitrile. Solvent B is a solution containing 0.1% formic acid and 90% acetonitrile. The tryptic peptides were dissolved in solvent A by liquid chromatography and separated using an EASY-nLC 1000 Ultra Performance Liquid Chromatography (UPLC) system. The specific gradient was comprised of an increase from 10 to 24% of solvent B over 26 min, 24–38% in 8 min (26–34 min), and climbing to 80% in 3 min (34–37 min), then holding at 80% for the last 3 min (37–40 min), and moving to a constant flow rate of 700 nL/min. The peptides were separated by an ultra-high performance liquid phase system, injected into an NSI ion source for ionization, and then analyzed by Orbitrap Fusion mass spectrometry. The primary mass spectral scan range is set to 350–1550 m/z, and the scan resolution is set to 60,000. The MS/MS scan range has a fixed starting point of 100 m/z, and the Orbitrap scan resolution is set to 15,000.

### Quantitative Real-Time PCR

Total RNA of peripheral blood mononuclear cells (PBMNCs) was extracted using TRIzol (Invitrogen, CA, United States). The purity and concentration of total RNA samples were quantified using the NanoDrop ND-1000 (NanoDrop Technologies/Thermo Scientific, Wilmington, DE, United States). Total RNA (2 μg) from each sample was reverse-transcribed using the Hairpin-it qRT-PCR kit (GenePharma, Shanghai, China) according to the manufacturer’s instructions. 20 μl reaction volume contained 10 μl Real-Time PCR Master Mix (SYBR), 0.4 μl 10 μM Gene-Specific Primer Set (0.2 μM final), 0.4 μl ROX reference dye (50×), 0.2 μl Taq DNA polymerase, and 2 μl of template cDNA and DEPC-treated water. GAPDH was used as an internal control to quantify and normalize the results. The 2^-△△CT^ value was used for comparative quantitation. All qRT-PCRs were performed in triplicate. The primers were obtained from GenePharma (Shanghai, China), and the sequences are shown in Supplementary Table S2.

### Western Blotting

Treated PBMNCs were lysed in RIPA buffer, and protein content was determined using a BCA Protein Assay Kit (cat. P0011, Beyotime). Fifty milligrams of protein samples was used for sodium dodecyl sulfate (SDS)/polyacrylamide gel electrophoresis (PAGE) and then transferred onto a PVDF membranes (Millipore, MA, United States). After blocking with 5% non-fat milk for 1 h, the membranes were incubated overnight at 4°C with the following primary antibodies: H4 (ab10158, Abcam, United Kingdom), H3.3C (ab150417, Abcam, United Kingdom), = two histone lysine acetylation site-specific antibodies, H3K23 acetyl antibody and H4K16 acetyl antibody (PTM Biolabs, Hangzhou, China), and GAPDH (cat. TA-08; ZSGB-BIO, China). After incubation with secondary antibodies (1:10,000), the specific bands were visualized by an electrochemiluminescence detection system. Relative protein content was analyzed by ImageJ software and normalized to loading controls.

### Cell Culture

INS-1 cells (rat insulinoma cells) were purchased from Enzyme Biotechnology (Shanghai, China). INS-1 cells were cultured in 15% fetal bovine serum, 10 mmol/L 2-[4-(2-hydroxyethyl)-1-piperazinyl] ethanesulfonic acid (HEPES), 100 U/ml penicillin, 100 μg/ml streptomycin, and 50 μmol/L β-mercaptoethanol in DMEM (low sugar) medium, at 37°C with 5% CO_2_. According to the experiment, INS-1 cells were cultured in 5.6 mmol/L glucose (normal glucose, NG group) and 33.3 mmol/L glucose (high glucose, HG group).

### Chromatin Immunoprecipitation Assay

INS-1 cells were fixed by 1% formaldehyde for 10 min at room temperature and lysed with ChIP lysis buffer (50 mM Tris HCl, pH 8.0, 5 mM EDTA, 1% SDS) for 10 min at 4°C. In a centrifuge tube, vortex, mix, and divide into multiple portions, each 500 uL, and place them in a 1.5 ml centrifuge tube for ultrasonic disruption. The ultrasonic power, ultrasonic time, interval time, and ultrasonic times are optimized. In the experiment, the ultrasonic power was set to 15 W (6%), 20 W (8%), 25 W (10%), and 30 W (12%); the ultrasonic time was set to 1, 3, and 5 s; the ultrasonic interval was set to 10, 20, 30, and 40 s; and the number of sonication was set to 15, 25, 30, and 40 times. When a certain condition is optimized, the other three conditions are fixed unchanged. The fixed value of each condition in this experiment is the ultrasonic power of 25 W (10%), the ultrasonic working time of 5 s, the interval of 40 s, and the ultrasonic wave of 30 times. After the samples were interrupted by an ultrasonic breaker, they were centrifuged at 10,000 r/min for 5 min at 4°C, and 400 uL was taken. The supernatant was aliquoted into two new centrifuge tubes, one for the uncross-linked chromosome assay and the other for the CHIP assay. Take 200 uL of ultrasonic sample and cross-linked sample, respectively, and add 8 uL of 5 mol/L. After incubation with NaCl for 6 h or overnight at 65°C, the cross-linked DNA and Hacl protein were de-cross-linked and then extracted with an extraction buffer. Extract once, then extract with chloroform. Add 20 g glycogen and 1 ml absolute ethanol to the extract to precipitate DNA for 1 h, and centrifuge at 13,000 r/min for 10 min at 4°C. Finally, 1 uL of RNase (10 mg/ml) was added and incubated at 37°C for 30 min. The above samples were subjected to 1% agarose gel electrophoresis to detect the ultrasonic effect of the samples to determine the optimized results of each condition. After sonication, the lysate was centrifuged, and the supernatant was collected and precleaned in dilution buffer (20 mM Tris HCl, pH 8.0, 2 mM EDTA, 150 mM NaCl, 1% Triton X-100) with protein G or A sepharose and salmon DNA for 2 h at 4°C. The precleaned samples were divided equally and incubated with the H4 antibody (ab10158, Abcam, United Kingdom) overnight at 4°C. Next, protein G or A sepharose was added into each sample and incubated for 2 h at 4°C. The beads were washed with TSE I, TSE II, buffer III, and tris-EDTA buffer sequentially. The samples were heated at 65°C for at least 6 h to reverse the cross-link. Then, DNA was purified by a PCR Cleaning Kit (Macherey-Nagel). Finally, the immunoprecipitated DNA was quantified by real-time PCR (Sybr green approach for heterochromatic loci).

### Immunofluorescence

INS-1 cells were seeded on a laser confocal culture dish and cultured in high glucose for 24–48 h. The cells were fixed (4% paraformaldehyde) for 30 min, washed 3 times with PBS, then permeabilized with 0.3% Triton X-100 for 20 min, and blocked with 1% BSA for 1 h. They were incubated overnight at 37.0°C in AcH4 (K16) and AcH3 (K23) antibodies (1:100). After washing, the sections were incubated with Alexa Fluor 488 goat anti-mouse IgG (Invitrogen, 1:1000) and Alexa Fluor 594 goat anti-rabbit IgG (Invitrogen; 1:1000) for 1 h at room temperature. Finally, add DAPI to stain the INS-1 cell nucleus (blue). The image was obtained by confocal (Leica, Germany).

### Data Analysis

The resulting MS/MS data were processed using the Maxquant search engine (v.1.5.2.8). Retrieval parameter settings: the database is SwissProt Human (20,317 sequences), an anti-library is added to calculate the false positive rate (FDR) caused by random matching, and a common contamination library is added to the database to eliminate contaminating proteins in the identification results. The effect of enzyme digestion is set to trypsin/P. The number of missed cleavage sites is set to four. The minimum length of the peptide is set to seven amino acid residues. The ion mass error tolerance was set at 20 and 5 ppm, respectively, and the mass error tolerance for secondary fragment ions was 0.02 Da. Cysteine alkylation was set as fixed modification, and variable modification as oxidation of methionine, acetylation of protein N-terminus, and acetylation of lysine. The quantification method was set to TMT-6plex, and the FDR of protein identification and PSM identification were both set to 1%. The cysteine alkylation was set as fixed modification, and variable modification as methionine oxidation, protein N-terminal acetylation, and lysine acetylation. The quantitative method is set to TMT-6plex, and the FDR for protein identification and PSM identification is set to 1%. Gene Ontology (GO) annotation proteome was derived from the UniProt-GOA database. If some identified proteins were not annotated, the InterProScan soft would be used to annotate protein’s GO functional based on the protein sequence alignment method. Annotate the identified proteins domain functional description using the InterPro domain database. Kyoto Encyclopedia of Genes and Genomes (KEGG) database was used to annotate the protein pathway. We used WoLF PSORT, a subcellular localization prediction soft, to predict subcellular localization. Soft MoMo (motif-x algorithm) was used to analyze the model of sequences constituted with amino acids in specific positions of modify-21-mers. The main software used in the analysis can be found in Supplementary Table S3.

### Statistical Analysis

In this study, the statistical analysis software used includes SPSS 19.0 and Origin 8.5. Quantitative values are expressed as mean ± standard deviation (SD), and statistical analyses were performed using Student’s *t*-test. All verification experiments (*e.g.,* qRT-RCR and western) require three biological replicates. Significance in statistical analysis was defined as *p* < 0.05.

## Result

### Modification Identification

**FIGURE 1:**
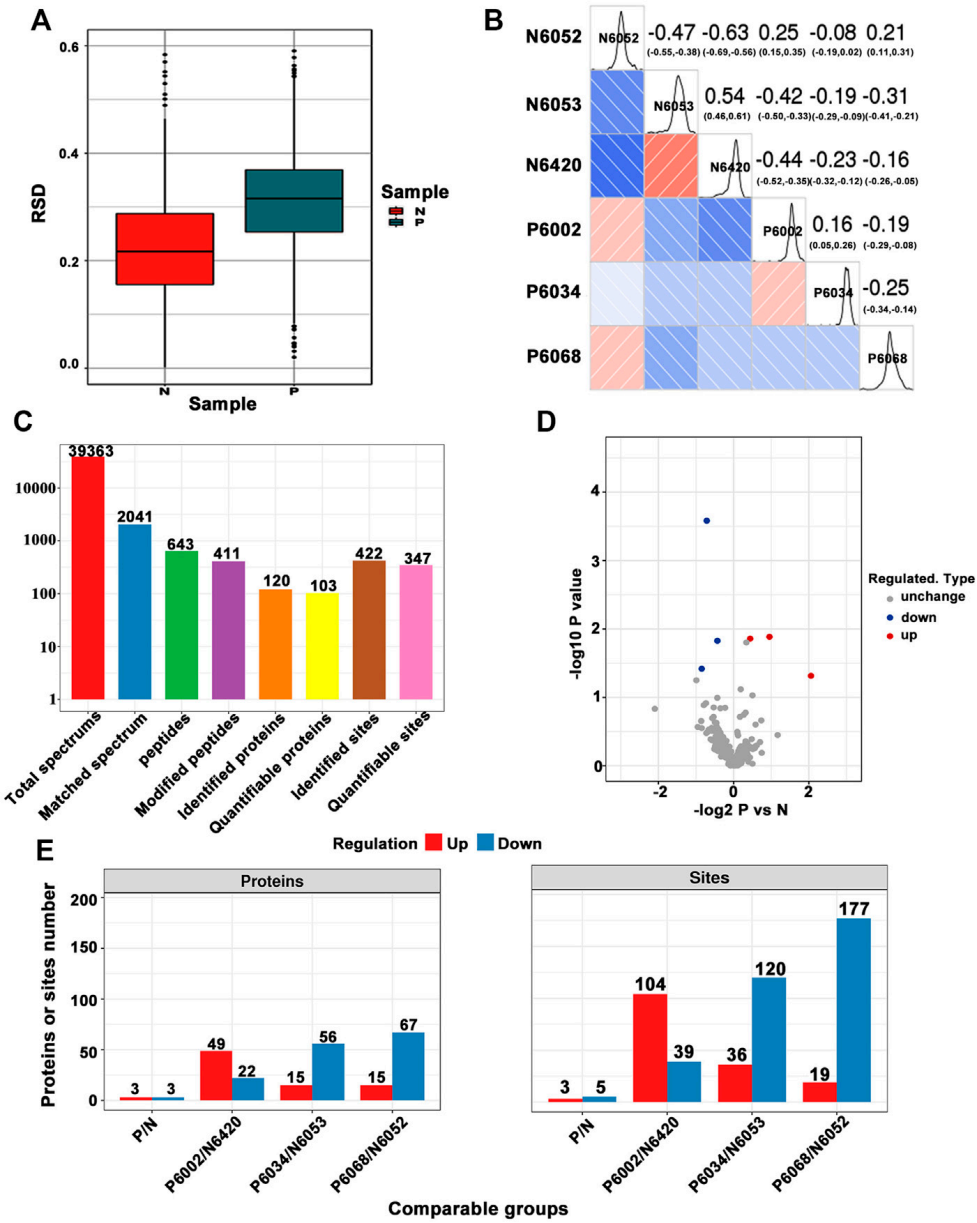
**(A)** Box plot of relative standard deviation (RSD) distribution of repeated samples using quantified proteins. **(B)** Heat map of Pearson’s correlation coefficients from all quantified proteins between each pair of samples. **(C)** Basic statistical figure of MS/MS results. **(D)** Volcano plot of differentially expressed modification sites. **(E)** Histogram of the number distribution of differentially expressed proteins and modification sites in different comparison groups.

A total of 39,363 MS/MS were obtained by LC-MS/MS. A schematic diagram of the technical route is shown in Supplementary Figure S1A. After searching by protein data, the available effective MS/MSs were 2041, and the utilization rate was 5.2%. In addition, 643 peptide fragments and 411 acetylated peptide fragments were identified by an effective MS/MSs analysis. Most of the peptides were distributed in 7–20 amino acids (Supplementary Figure S1B), consistent with the general rule based on trypsin enzymatic hydrolysis and high energy collision-induced dissociation (HCD) fragmentation. Among them, peptide fragments of less than five amino acids cannot produce effective sequence identification due to the small number of fragment ions generated. Peptides larger than 20 amino acids were not suitable for fragmentation of HCD due to their high mass and charge. The distribution of peptide lengths identified by mass spectrometry meets the quality control requirements. The number of modification sites corresponding to each protein is shown in Supplementary Figure S1C, and the mass precision map of the MS/MSs data is shown in Supplementary Figure S1D. The SDS-PAGE of protein extraction quality control is shown in Supplementary Figure S2, and the initial protein concentration determination results of each sample are shown in Supplementary Figure S4. Taken together, according to protein extraction quality control standards, our protein extraction quality meets the requirements. Herein, we use two statistical analysis methods, relative standard deviation (RSD, Figure 1A) and Pearson’s correlation coefficient (Figure 1B), to evaluate sample repeatability. In order to ensure a high degree of credibility of the results, we used the standard of localization probability >0.75 to filter the authentication data. One is the highest score. In total, 422 acetylation sites from 120 acetylated proteins were identified, of which 347 sites on 103 proteins have quantitative information (Figure 1C).

### Differential Protein Modification Analysis

In this study, quantitatively repeat the experiment through three modifications to obtain the quantitative value of each sample in the three replicates. Firstly, we calculated and compared the differential modification of the modification sites between two samples in the group. Each sample is repeated three times to obtain the average value of quantitative values and then calculated for the ratio of the average value between the two samples, which is used as relative quantification (ratio) of final differential modification of group comparison. Secondly, we calculated the significant *p*-value of the different modifications. Taking the quantitative value of each sample as log_2_ so that the data conforms to the normal distribution, we then calculated the *p*-value using the two-sample *t*-test method (two-tailed). When *p*-value < 0.05, the difference modification amount is more than 1.3 as a significant upregulation change threshold, and less than 1/1.3 is a significant downregulation. According to screening criteria, we construct a volcano plot of differentially expressed modification sites. In Figure 1D, six differentially modified proteins have been marked (as blue and red circles). Among them, compared with the control group, we found that the modification levels of the three sites were upregulated, and the five sites were downregulated (Table 1 for details). The specific distribution of differentially expressed proteins and modification sites in different comparison groups is shown in Figure 1E. Among them, each sample was subjected to three technical repetitions.

**TABLE 1:** Differentially expressed proteins and modification sites (P/N).

Protein accession	Position	P/N ratio	Regulated type	Amino acid	P/N *p* value	Protein description	Gene name	Score
P01042	476	4.158	Up	K	0.0484	Kininogen-1	KNG1	159.26
P01860	218	0.555	Down	K	0.0381	Immunoglobulin heavy constant gamma 3	IGHG3	143.93
P04114	51	1.356	Up	K	0.0138	Apolipoprotein B-100 (ApoB-100)	APOB	121.25
P05155	316	1.935	Up	K	0.013	Plasma protease C1 inhibitor	SERPING1	55.261
P62805	13	0.609	Down	K	0.0003	Histone H4	HIST1H4A	113.62
P62805	17	0.609	Down	K	0.0003	Histone H4	HIST1H4A	113.62
Q6NXT2	24	0.741	Down	K	0.0149	Histone H3.3C	H3F3C	155.11
Q6NXT2	19	0.741	Down	k	0.0149	Histone H3.3C	H3F3C	155.11

### Protein Annotation

**FIGURE 2:**
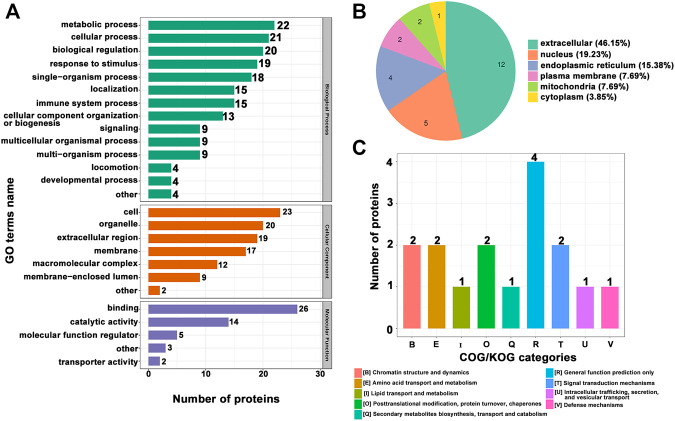
**(A)** Statistical distribution chart of proteins corresponding to differentially expressed modification sites under each GO category (second level). **(B)** Subcellular localization chart of proteins corresponding to differentially expressed modification sites. **(C)** COG/KOG functional classification chart of proteins corresponding to differentially expressed modification sites.

In order to thoroughly understand the modified proteins identified and quantified in the data, we detailed the functions and characteristics of these proteins from the aspects of GO, protein domain, KEGG pathway, and subcellular structure localization. The GO annotations are divided into three broad categories, biological process (BP), cellular component (CC), and molecular function (MF), which explain the biological effects of proteins from different perspectives. We performed statistics on the distribution of proteins corresponding to differentially expressed modification sites under each GO category (second level), as shown in Figure 2A. Moreover, we constructed a subcellular localization chart of proteins corresponding to differentially manifest modification sites (Figure 2B). Clusters of Orthologous Groups (COG) of proteins is a database of NCBI. COG for eukaryotic complete genomes is called the KOG. The proteins corresponding to differentially expressed modification sites were subjected to COG/KOG functional classification statistics by database alignment analysis (Figure 2C).

### Affinity Enrichment

**FIGURE 3:**
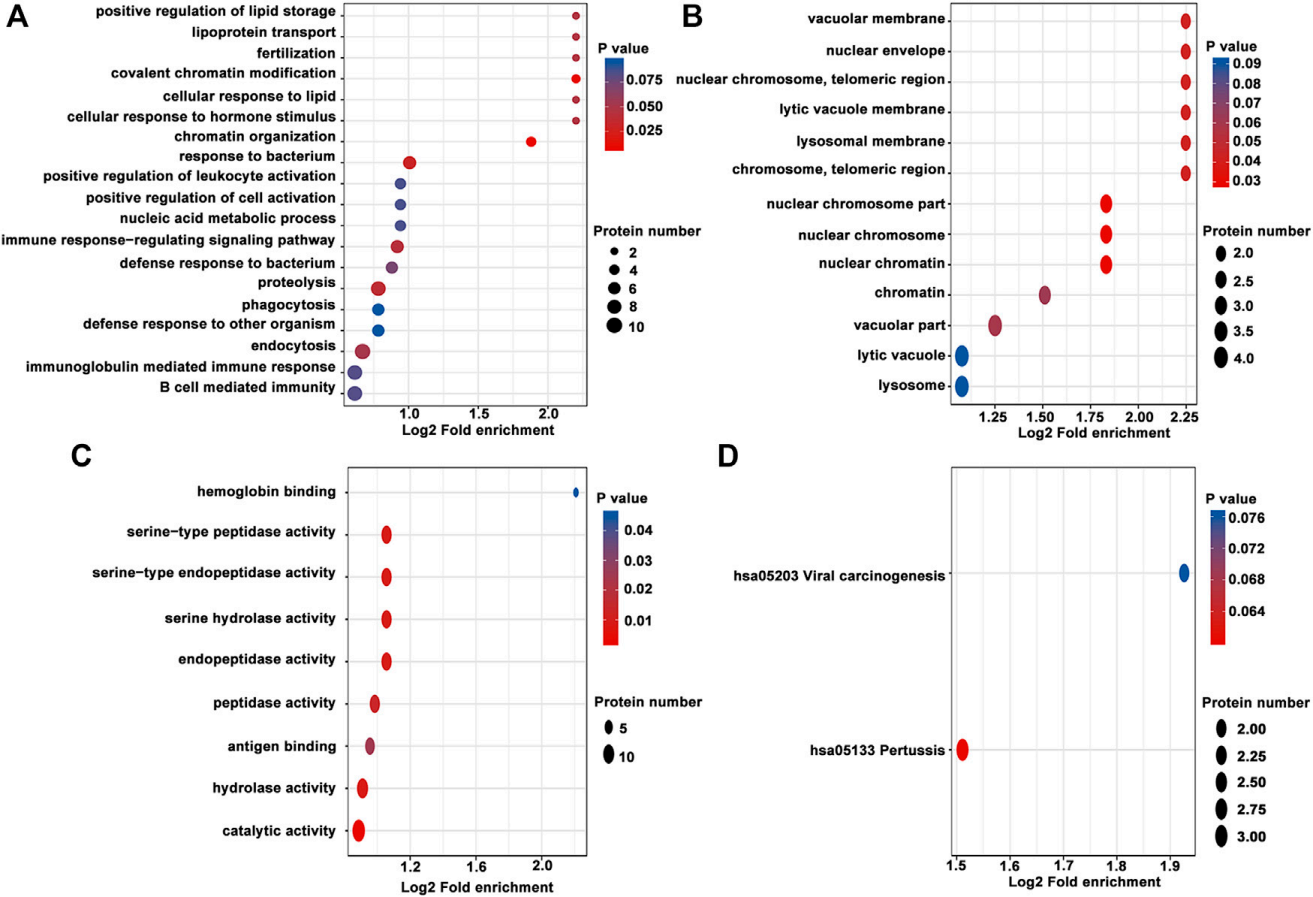
GO enrichment bubble plot of proteins corresponding to differentially expressed modification sites in three categories. **(A)** Biological process. **(B)** Cellular component. **(C)** Molecular function. **(D)** KEGG pathway enrichment bubble plot of proteins corresponding to differentially expressed modification sites.

**FIGURE 4:**
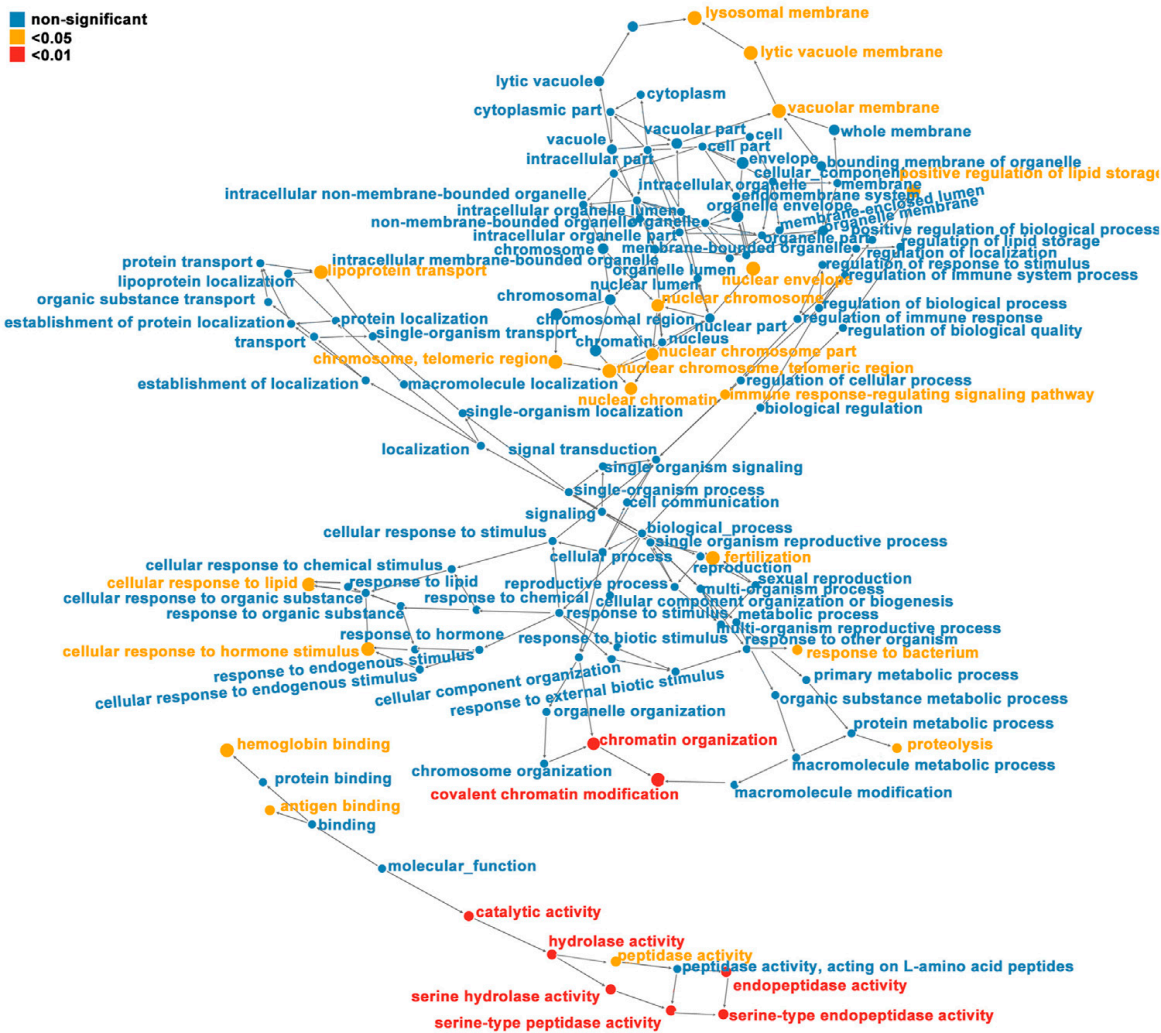
Directed acyclic graph (DAG) of each GO category for proteins corresponding to differentially expressed modification sites (red indicates that the differentially modified protein is extremely significant (*p* < 0.01) enriched in GO classification, yellow represents differentially modified protein (*p* < 0.05) enriched GO classification, and blue represents non-significantly enriched GO classification. The upper and lower levels of the GO classification; the size of the circle represents the degree of enrichment.

For enrichment, the *p*-values obtained by Fisher’s exact test were used to construct a bubble diagram to reveal the functional classification and pathway for significant enrichment of differentially modified proteins (*p* < 0.05). Therefore, we drew a GO enrichment bubble plot of proteins corresponding to differentially expressed modification sites in three categories (Figures 3A–C). Similarly, we constructed a KEGG pathway enrichment bubble plot of proteins corresponding to differentially manifest modification sites (Figure 3D). The protein domain enrichment bubble plot of proteins corresponding to differentially expressed modification sites is shown in Supplementary Figure S3. In the bubble chart, the circle color indicated the *p*-value of the enrichment significance, and the circle size indicated the number of differentially modified proteins in the functional classification or pathway. Furthermore, the directed acyclic graph (DAG) of each GO category for proteins corresponding to differentially expressed modification sites is shown in Figure 4.

### KEGG Pathway

**FIGURE 5:**
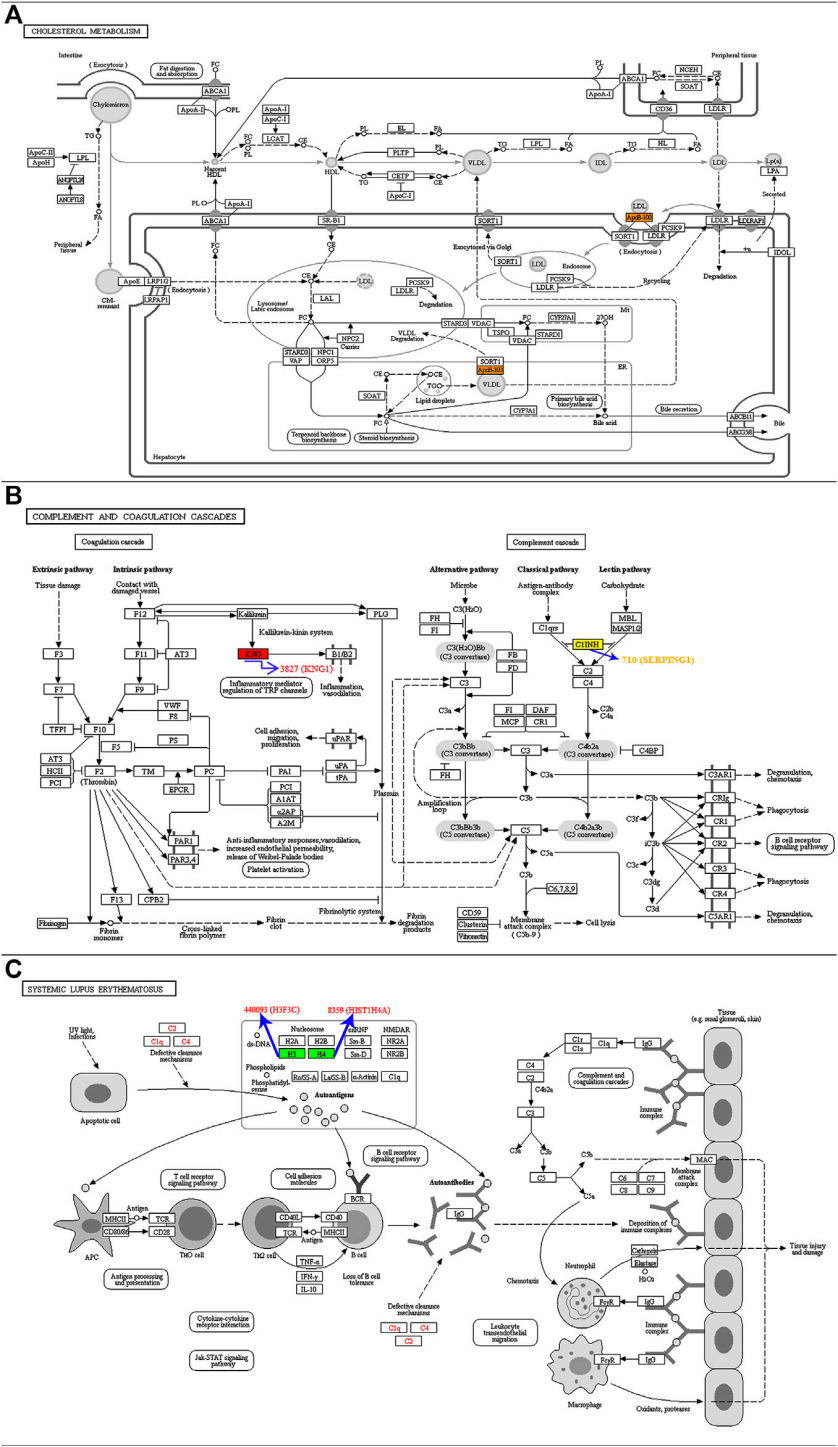
KEGG pathway map showing significant enrichment of proteins corresponding to differentially expressed modification sites. **(A)** ApoB-100 protein was enriched into the has04979 pathway. **(B)** KNG1 and SERPING1 proteins were enriched into the has04610 pathway. **(C)** Histones H4 and H3.3C were enriched into the has05322 pathway.

KEGG is an information network that links intermolecular interactions. Combining our previous results (Table 1), we performed significant KEGG pathway enrichment on proteins corresponding to differentially expressed modification sites to help us understand the function and role of protein acetylation in T2DM in depth. Apolipoprotein B (ApoB-100) protein corresponding to differential acetylation site was enriched into the cholesterol metabolism pathway (hsa04979, Figure 5A). Proteins of kininogen-1 (KNG1) and plasma protease C1 inhibitor (SERPING1) corresponding to differential acetylation sites were enriched into the complement and coagulation cascades pathway (hsa04610, Figure 5B). Histones H4 and H3.3C corresponding to differential acetylation sites were enriched into the systemic lupus erythematosus pathway (hsa05322, Figure 5C).

### Cluster Analysis

**FIGURE 6:**
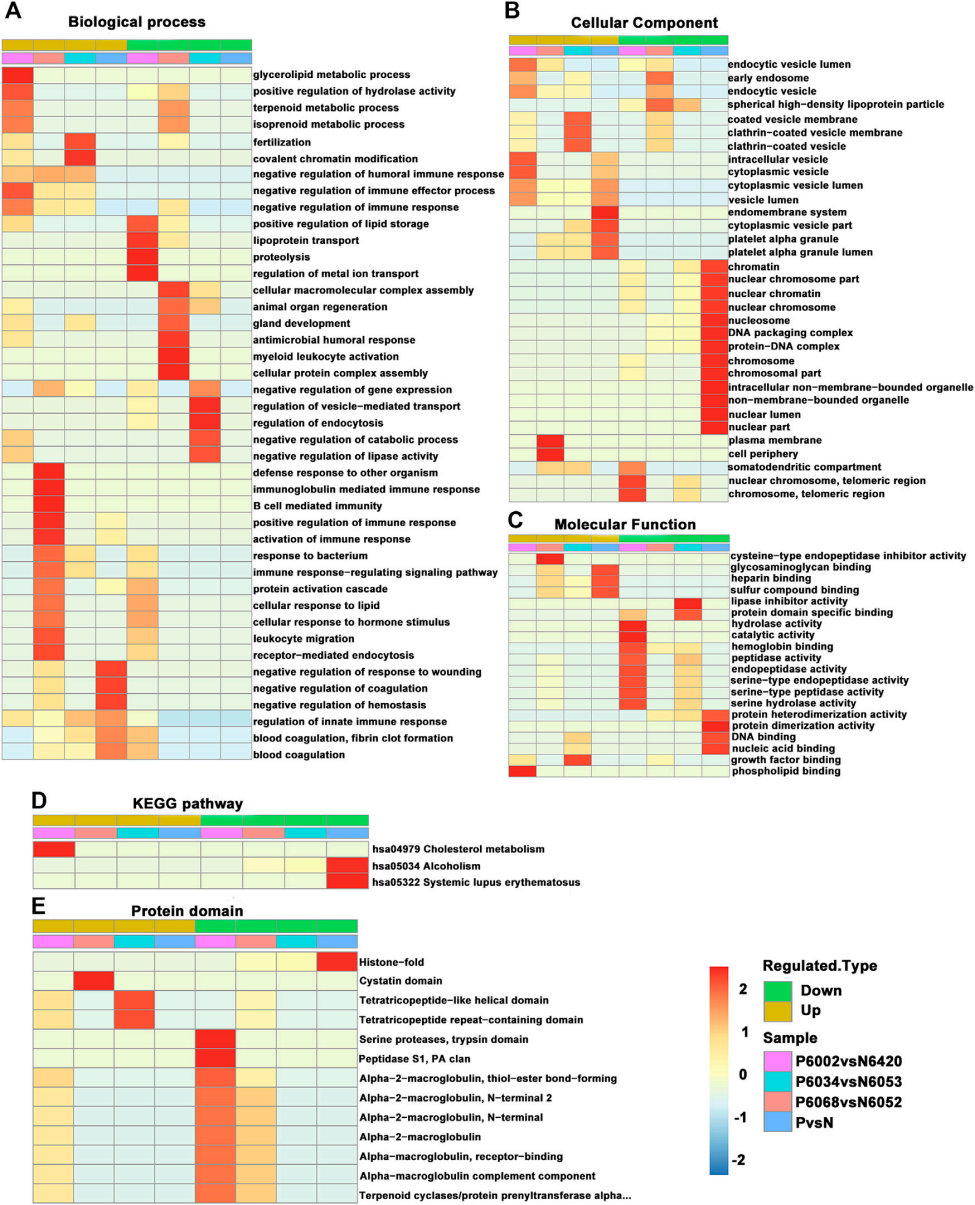
A comprehensive heat map for cluster analysis of the enrichment patterns of GO functional categories, KEGG pathways, and protein domains. **(A)** Biological process. **(B)** Cellular component. **(C)** Molecular function. **(D)** KEGG pathway. **(E)** Protein domain.

Cluster analysis of differentially modified proteins in different comparison groups was performed to find the correlation of the function of differentially modified proteins (Figure 6). The specific method was to use hierarchical clustering to collect related functions in different groups according to the *p*-value of the enrichment test (Fisher’s exact test) and draw it as a heat map. In addition, the horizontal direction of the heat map represents different comparison groups, and the vertical direction is a description of the differential modification-related function (GO, KEGG pathway, protein domain). Different sets of differentially modified proteins and their functional descriptions correspond to the color blocks, suggesting the strength of different enrichment levels. Among them, red indicates strong enrichment and blue indicates weak enrichment.

### Protein Motif Analysis

By counting the regularity of upstream and downstream amino acids of all acetylation modification sites in the sample, the regular trend of the amino acid sequence in the acetylation site was calculated. However, it is a pity that we did not find enrichment to the modified site feature sequence (Supplementary Figure S4).

### Protein Interaction Network

**FIGURE 7:**
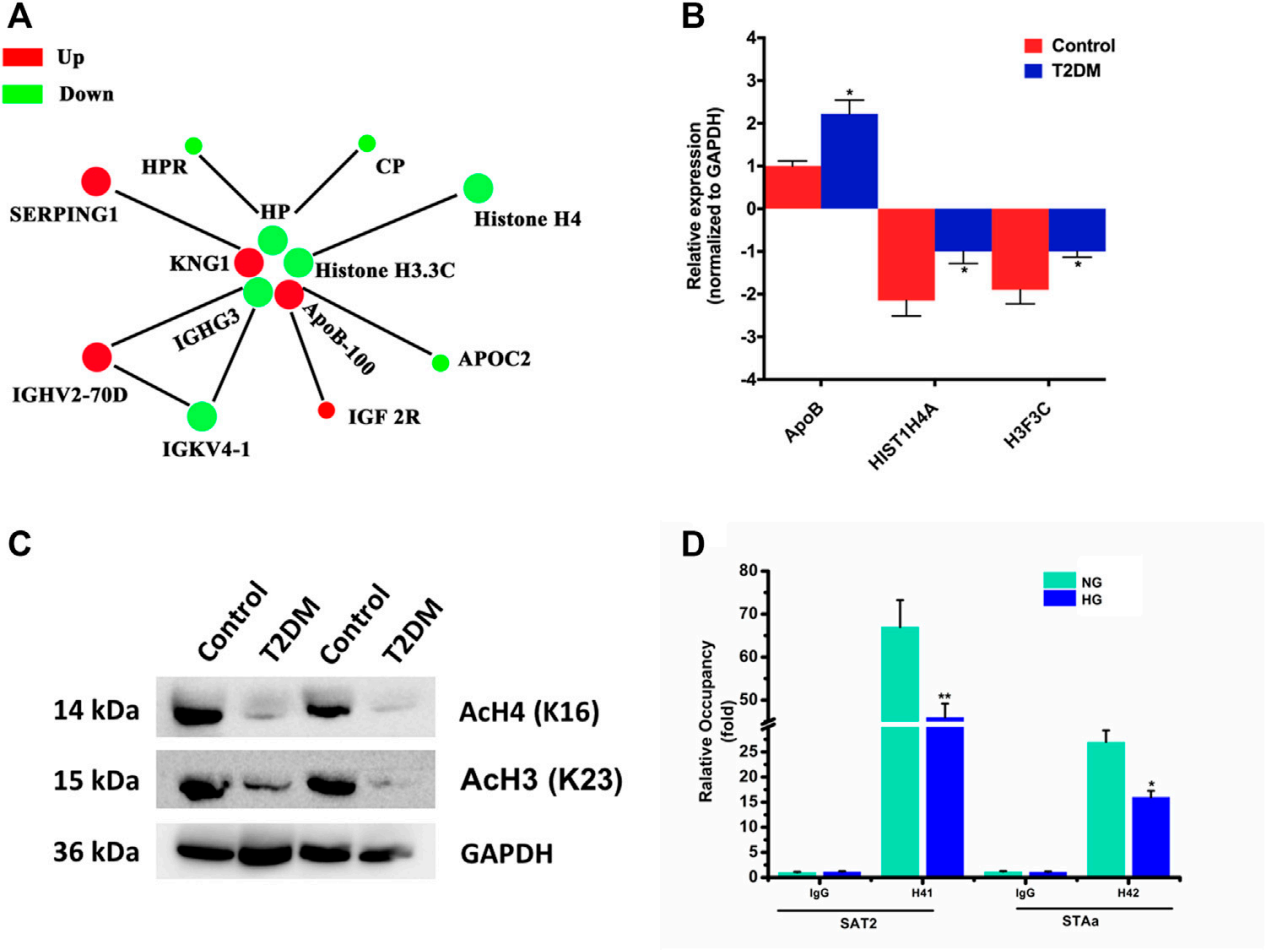
**(A)** Protein–protein interaction network for proteins corresponding to differentially expressed modification sites. Different colors represent differential modifications of the protein (green is the downregulated and red is the upregulated). **(B)** Proteins corresponding to differentially expressed modification sites validated by qRT-PCR. All PCRs were performed in triplicate. The data are represented as means ± SD. **(C)** Western blot detection of H4K16 and H3.3CK23 acetylation levels between Uyghur T2DM and the control group. **(D)** Using ChIP to detect H4 acetylation levels of SAT2 and STAa in INS-1 cells of different groups. *p < 0.05, **p < 0.01.

The differentially modified protein database number or protein sequence screened in different groups were compared with the STRING (v.10.5) protein network database, and the differential protein interaction relationships were extracted according to the confidence score >0.7 (high confidence). Then, visualize the differential protein interaction network using the R package “networkD3” tool (Figure 7A). The circle size represents the number of differentially modified proteins and their interacting proteins. The larger the circle, the more proteins it interacts with, indicating the more important the protein in the network.

### Verification of Proteins Corresponding to Differentially Expressed Modification Sites

We selected ApoB-100 and histones H4 and H3.3C for the level of mRNA detection by qRT-PCR. For verification, we collected 60 cases of Uyghur T2DM subjects and normal individuals, respectively (the same batch as proteomic analysis). Moreover, we detected differentially expressed modification sites in PBMNCs. The result of qRT-PCR in T2DM patients demonstrated that they were consistent with the proteomic analysis (Figure 7B). Then, we detected the protein levels of histones H4 and H3.3C by western blot. The results showed that the protein levels of H4 and H3.3C in T2DM patients decreased compared to normal controls (Supplementary Figure S5). Subsequently, we conducted a specific detection of the acetylated protein levels at the H4K16 and H3K23 sites between Uyghur T2DM and the control group (Figure 7C). The results showed that compared with the control group, the H4K16 and H3.3CK23 acetylation levels in Uyghur T2DM were significantly lower. In addition, a quantitative ChIP assay also revealed that acetylated H4 was downregulated in both SAT2 and SATa loci in INS-1 cells with high glucose (Figure 7D). Taken together, these provide a theoretical basis for us to understand the protein acetylation status of Uyghur patients with T2DM.

### Immunofluorescence Analysis of AcH4 (K16) and AcH3 (K23) in INS-1 Cells

**FIGURE 8:**
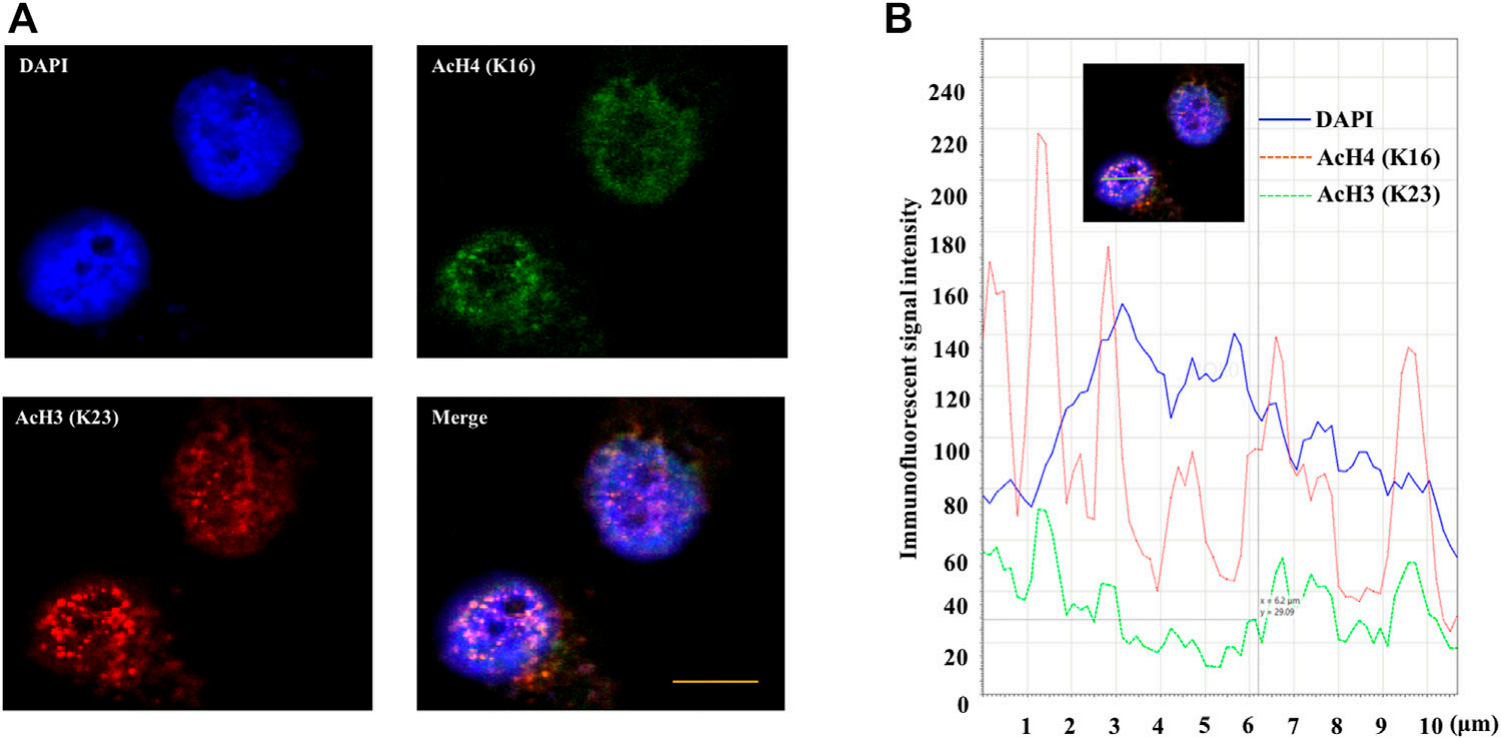
Double immunofluorescence staining of AcH4 (K16) and AcH3 (K23) in high glucose cultured INS-1 cells. **(A)** Double immunofluorescence staining image of AcH4 (K16) and AcH3 (K23) in INS-1 cells. **(B)** The distribution of immunofluorescence signal intensity in the nucleus. Use LAS X Life Science to perform image analysis on the merged image. The nucleus area is shown by the green bar. Scale bar: 10 μm.

The double immunofluorescence staining results of AcH4 (K16) and AcH3 (K23) in high glucose cultured INS-1 cells are shown in Figure 8A. These two modifications are detected as distinct punctate regions in the nucleus. AcH4 (K16) is mainly observed in the peripheral heterochromatin area, while AcH3 (K23) can be observed in the peripheral heterochromatin area and the central euchromatin area of the nucleus. Quantitative intensity analysis using LAS X Life Science image analysis software confirmed the different immunofluorescence intensity peaks between AcH4 (K16) and AcH3 (K23) (seen in Figure 8B).

## Discussion

This article aims to provide a solid foundation for further research on protein acetylation in Uyghur patients with T2DM. By screening criteria, we found that proteins of ApoB-100, KNG1, and SERPING1 corresponding to differentially expressed modification sites were upregulated. Studies have demonstrated that ApoB has a certain correlation with metabolic syndrome and insulin resistance. ApoB could be incorporated into communicating increased risk for diabetes, in addition to the progression of atherosclerotic disease, especially among high-risk populations (Ley et al., 2010). The current European guidelines also recommend using non-HDL-C and Apo-B as metabolic syndrome, diabetes, high triglyceride levels, or chronic kidney disease (European Association for Cardiovascular et al., 2011). The fasting concentration of ApoB-100 has been related to the severity of angiographic findings in diabetic patients (Tkac et al., 1997). Herein, we found that the acetylation level of ApoB-100 protein K51 (ApoB-100K51) in T2DM patients was significantly upregulated, suggesting that ApoB-100K51 acetylation may be involved in the development of T2DM.

Moreover, kininogen fragments deserve special attention as the kallikrein–kinin pathway is considered to play a central role in diabetic nephropathy (DN) pathogenesis (Vitova et al., 2017). Although the exact function of the kallikrein–kinin system in the pathogenesis of DN has not been fully elucidated, it is considered to play a beneficial role (Tomita et al., 2012). KNG1 were downregulated consistently in T1DM but upregulated in T2DM (Zhang et al., 2013). In addition, the main function of SERPING1 is to inhibit the complement system from preventing spontaneous activation, which is an acute-phase reaction protein (Davis, 2004). In some existing proteomics studies, SERPING1 was associated with insulin resistance (Zhang et al., 2013). Our study displayed that the acetylation levels of SERPING1 K316 and KNG1 K476 sites were upregulated and may also provide a basis for the involvement of KNG1 and SERPING1 in the pathogenesis of T2DM.

The levels of acetylation modification at five sites in the differential protein were downregulated, and we are very interested in histones H4 and H3.3C. Histone acetylation can affect the activation and silencing of gene transcription by dynamically regulating the structure of chromatin (Du et al., 2015). Histone acetylation is highly reversible and dynamic, which can be catalyzed by histone acetyltransferases (HATs) or histone deacetylases (HDACs), respectively. Histone acetylation on H3 and H4 has been considered as a marker of an “open” configuration of chromatin. HATs and HDACs have been shown to regulate gene expression associated with diabetes (Miao et al., 2004; Christensen et al., 2011). The hallmark of insulin gene activation is high levels of H3Ac and H3K4Me in its promoter region (Chakrabarti et al., 2003). Upregulation of H3K9 acetylation levels indirectly promotes islet beta-cell proliferation (Kawada et al., 2017). Recently, HDAC inhibitors have been found to improve insulin resistance in peripheral tissues, thereby reducing inflammation and improving and delaying diabetes complications (Ye, 2013). The above results confirmed that the dysregulation of histone acetylation was closely related to the occurrence of diabetes. The acetylation levels of histones H4K13, H4K16, H3.3CK19, and H3.3CK23 were significantly downregulated in T2DM patients, and this may indicate that these histone acetylation levels in the normal population can promote gene transcription, thereby decreasing blood glucose. However, considering the dynamic reversibility of histone acetylation (Turner, 2000; Sims and Reinberg, 2008), it may also indicate an increase in these histone deacetylation levels, inhibiting the transcription of genes and participating in the elevation of blood glucose. There is also a possibility that it cannot be ignored. Uyghur patients with T2DM stimulate histone modification enzymes due to elevated blood glucose *in vivo*, causing changes in the levels of acetylation and deacetylation of H4K13, H4K16, H3.3CK19, and H3.3CK23. This also provides a basis for the pathogenesis of T2DM in Xinjiang Uyghur and also provides a new direction for the development of diabetes drugs.

The distribution of the protein corresponding to the differential modification site in the GO secondary annotation was constructed. Among them, the metabolic process, response to stimulus, immune system process, cellular component organization, or biogenesis were all highly correlated with diabetes. For the KEGG pathway, ApoB-100 protein was enriched into the hsa04979 pathway. KNG1 and SERPING1 proteins were enriched into the hsa04610 pathway. KNG1 participates in the coagulation cascade in the pathway, and SERPING1 participates in the complement cascade. In addition, KNG1 and SERPING1 proteins have a close interaction network relationship. Histones H4 and H3.3C were enriched into the hsa05322 pathway. The hsa05322 pathway involves not only the TNF-α and JAK-STAT pathway but also the complement and coagulation cascade. It is well known that most inflammatory factors play an important role in the development and progression of T2DM, such as TNF-α and interleukin. Studies have displayed that DNA methylation in the promoter region alters the expression of TNF-α and other genes in visceral adipose tissue of Uyghur patients with diabetes (Zhang et al., 2017). High glucose culture conditions can cause elevated levels of histone acetylation in the promoter region of inflammatory genes such as TNF-α (Miao et al., 2004). Furthermore, histones H4 and H3.3C have a close interaction network. Therefore, how the acetylation modification of H4K13, H4K16, H3.3CK19, and H3.3CK23 affects the hsa05322 pathway is worthy of further study.

For the verification of the proteins corresponding to the differentially expressed modification sites, we selected ApoB-100 and histones H4 and H3.3C to detect mRNA and total protein levels for verification. Our verification results showed that the mRNA and total protein expression levels of ApoB-100 and histones H4 and H3.3C are significantly different between the two groups. In addition, we further carried out specific detection of the acetylated protein levels at H4K16 and H3K23 sites between Uyghur T2DM and the control group. The results demonstrated that compared with the control group, the acetylation levels of H4K16 and H3K23 in Uyghur T2DM were significantly reduced. Taken together, our preliminary screening and verification from the mRNA and acetylated protein levels have shown that it is consistent with the quantitative study of acetylated proteomics.

## Conclusion

T2DM is a multi-gene and multi-factor disease. It is difficult to fully reveal the molecular mechanism of disease occurrence by studying the epigenetic modification of a single gene. The imbalance of multi-gene apparent modification may play an important role in the pathogenesis of T2DM. Our research has concentrated on proteomic analysis of lysine acetylation sites modifications to provide a reliable theoretical basis for clinical treatment in Uyghur patients with T2DM. In addition, a comprehensive understanding of histone acetylation mechanisms can give rise to novel therapeutic options for T2DM, and histone acetylation regulation could be promising therapeutic targets for T2DM. Utilizing precise inhibitors of histone-modifying enzymes, this may represent a new strategy to prevent T2DM.

## Data Availability

The datasets presented in this study can be found in online repositories. The names of the repository/repositories and accession number(s) can be found in the article/Supplementary Material.
