# Supplementary figures and images for: Proteomic Analysis of Serum Lysine Acetylation in Uyghur Patients With T2DM
Source: Front Mol Biosci. 2022 Mar 30;9:787885. doi: 10.3389/fmolb.2022.787885 (PMC9006524; doi:10.3389/fmolb.2022.787885)

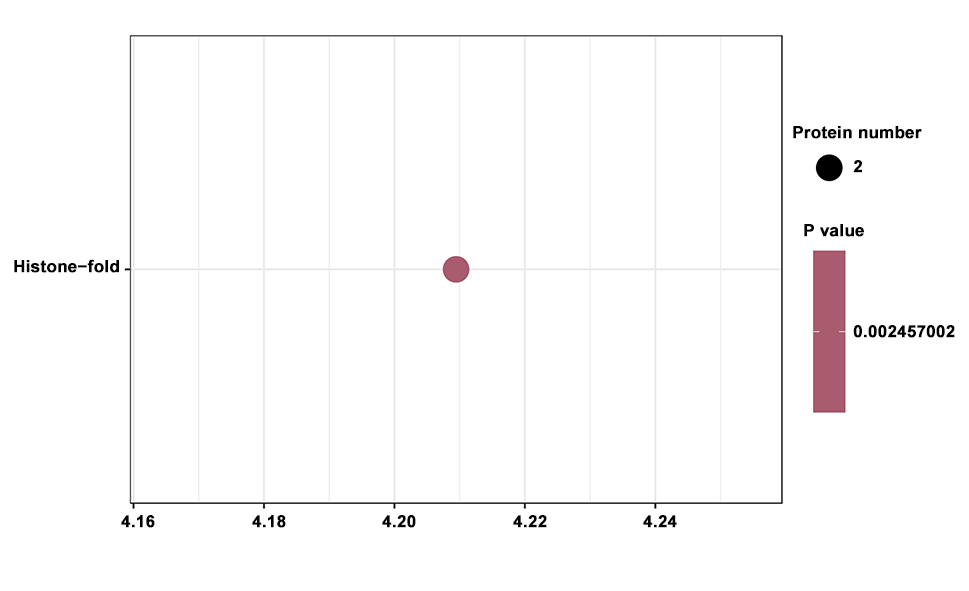

Supplement: Supplementary file 1 [file Image3.TIFF]

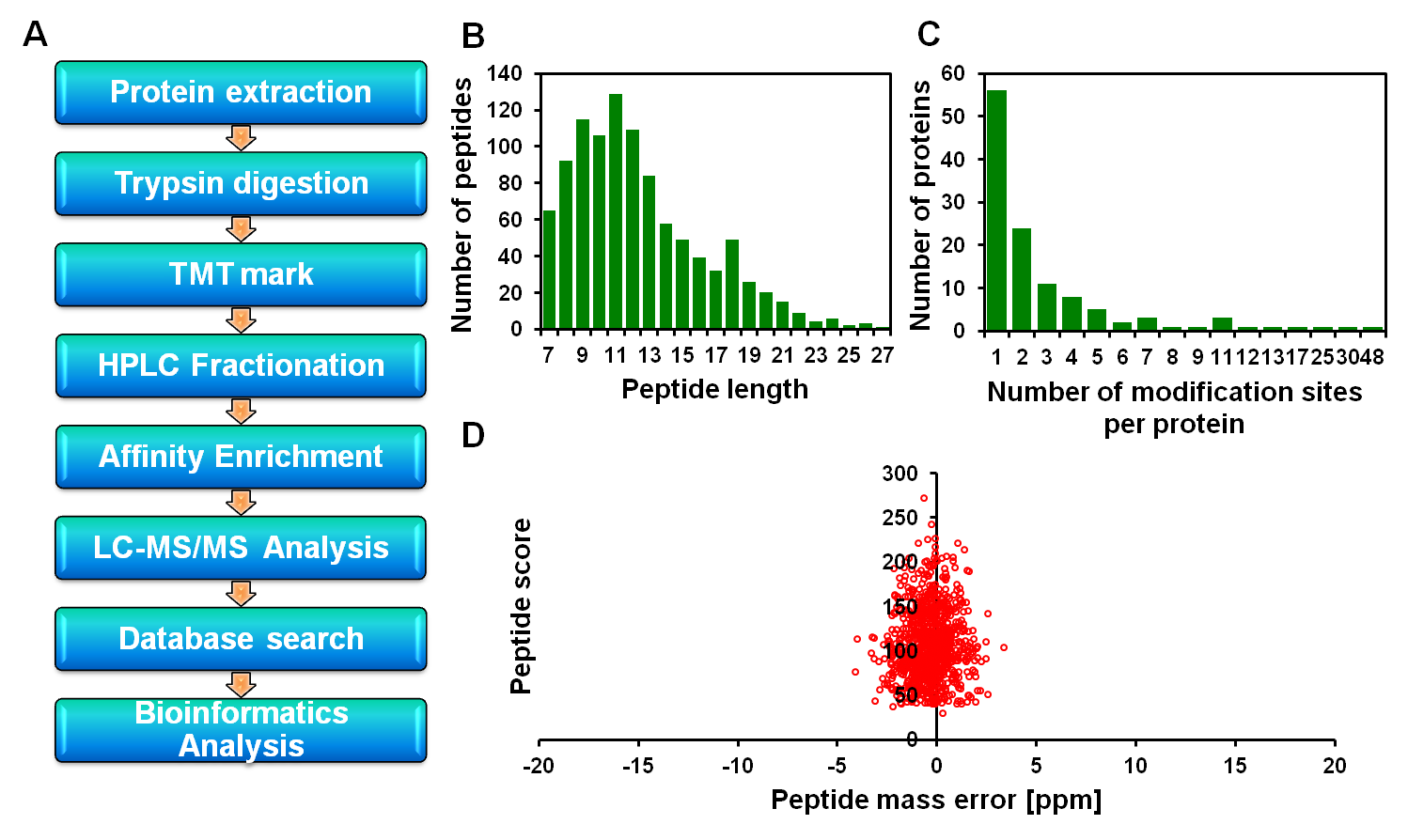

Supplement: Supplementary file 2 [file Image1.TIFF]

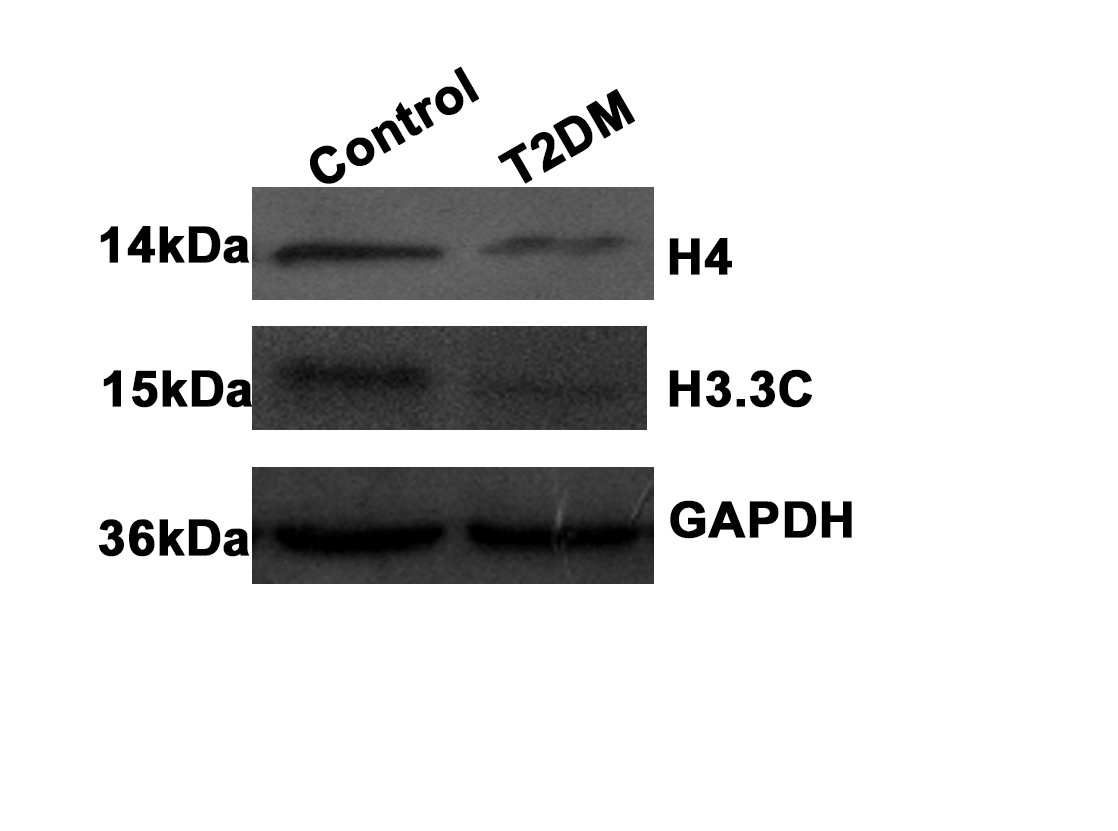

Supplement: Supplementary file 3 [file Image5.PNG]

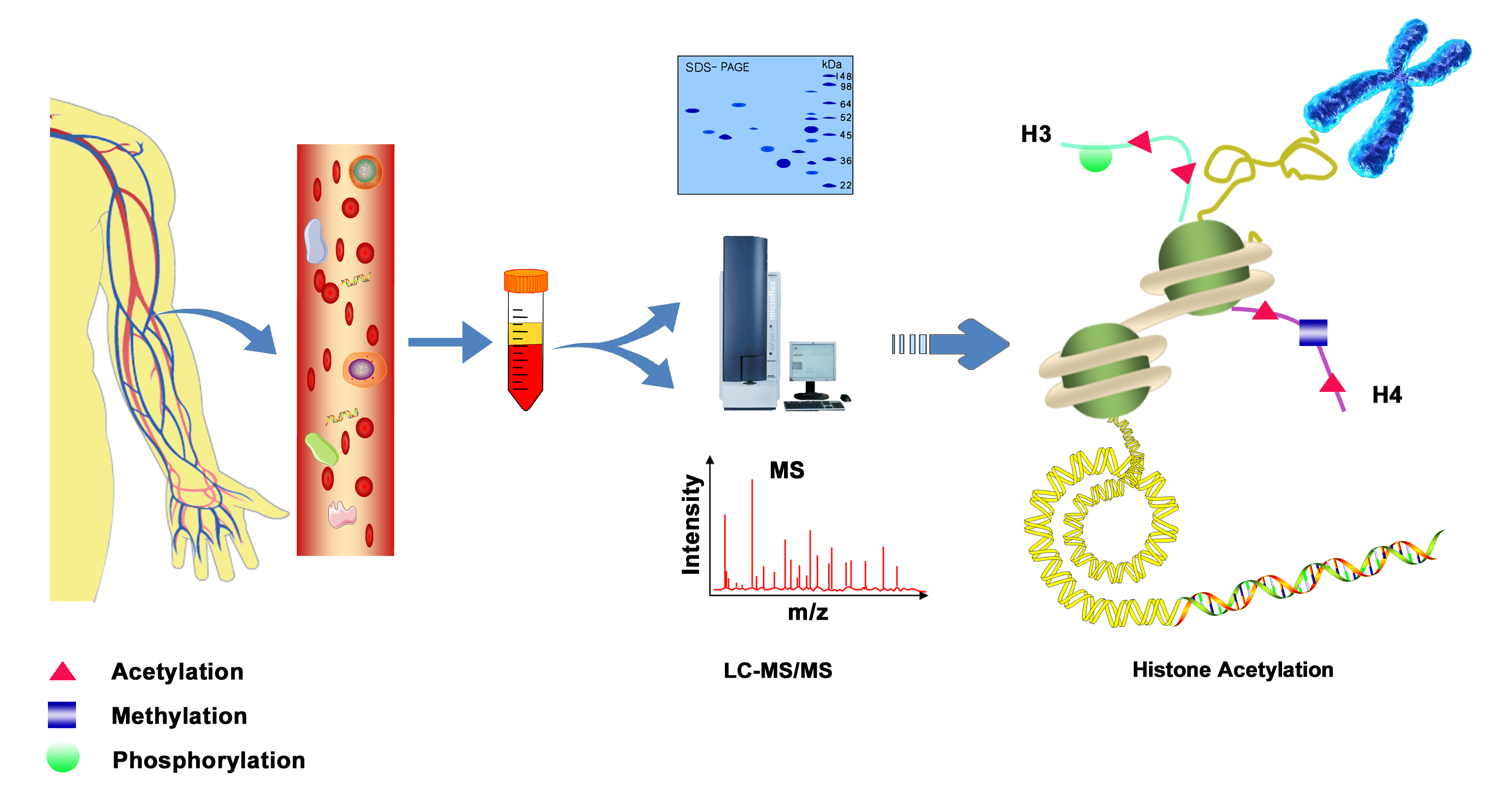

Supplement: Supplementary file 4 [file Image6.TIFF]

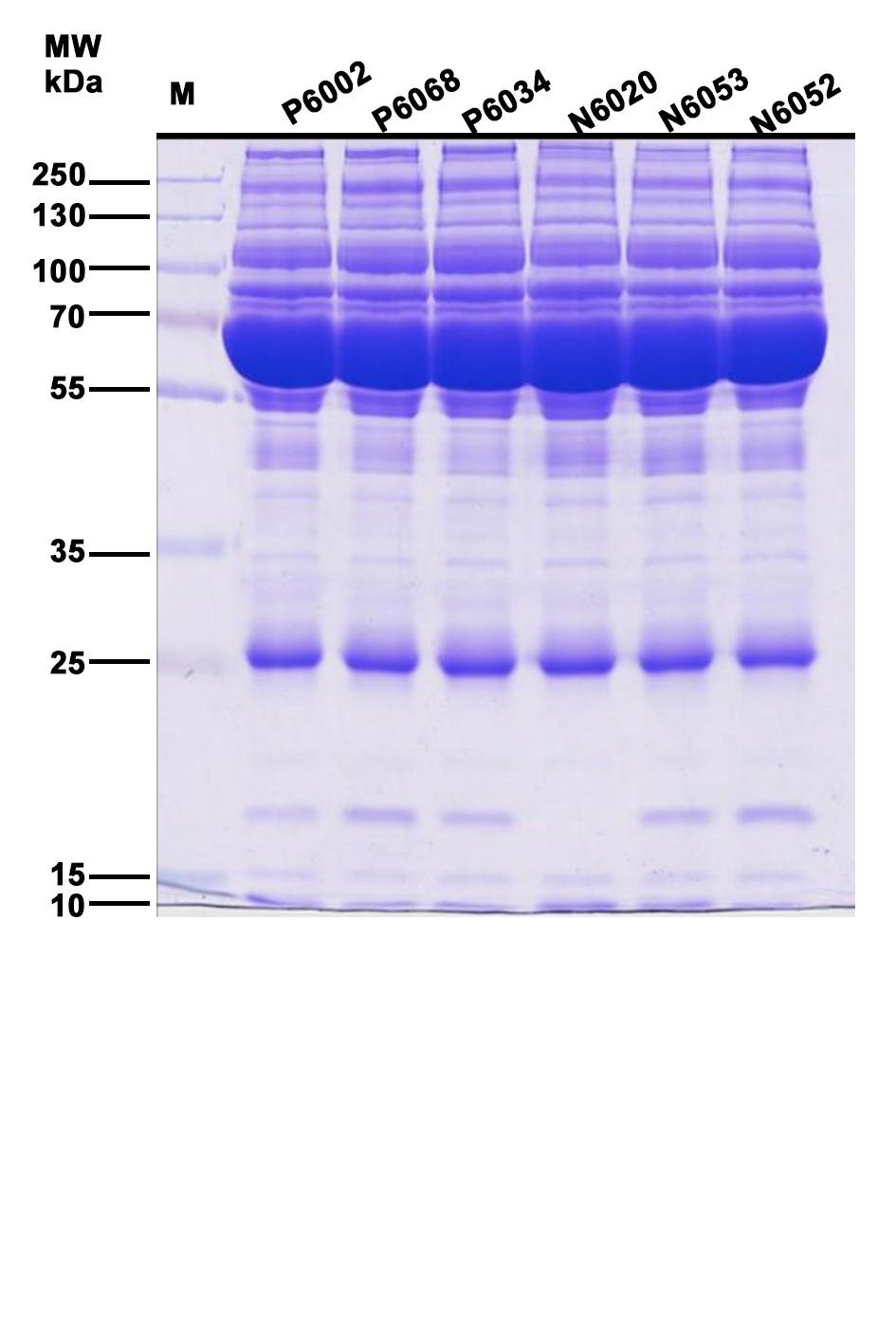

Supplement: Supplementary file 5 [file Image2.TIFF]

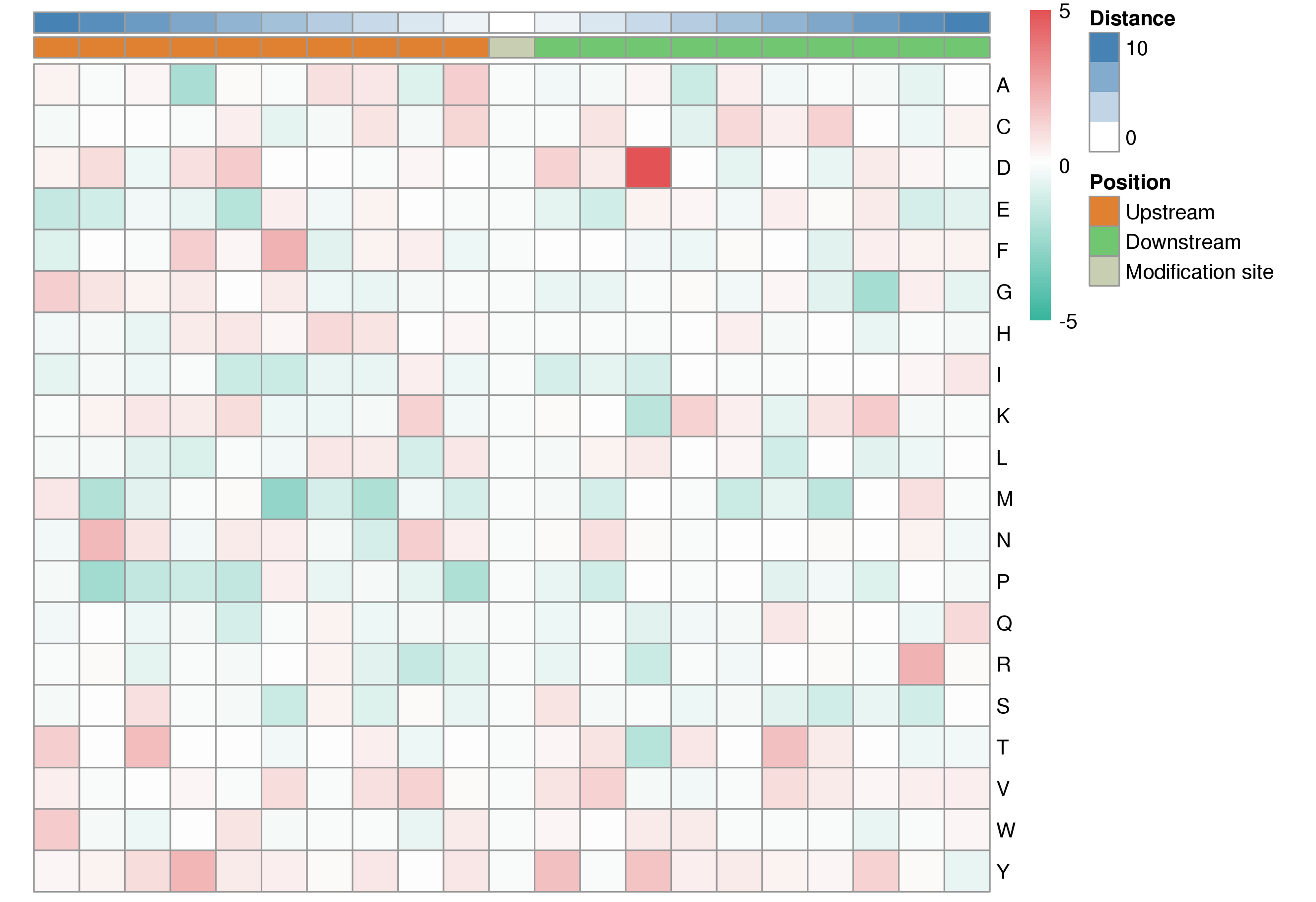

Supplement: Supplementary file 6 [file Image4.TIFF]
