# Supplementary material for: Proteomic Analysis of Serum Lysine Acetylation in Uyghur Patients With T2DM
Source: Front Mol Biosci. 2022 Mar 30;9:787885. doi: 10.3389/fmolb.2022.787885 (PMC9006524; doi:10.3389/fmolb.2022.787885)
Supplement: Supplementary file 7 [file DataSheet1.docx]

**Supplementary Material**

**Figures legends and Tables**

**Fig. S1** **A**, Schematic diagram of the technical route; **B**, Length distribution of peptides identified by MS/MS. **C**, The number of modification sites corresponding to per protein. D, The mass precision distribution of MS/MSs data.

**Fig. S2** SDS-PAGE of protein extraction quality control, with 30 μg loaded on each lane.

**Fig. S3** Protein domain enrichment bubble plot of proteins corresponding to differentially expressed modification sites.

**Fig. S4** The motif enrichment heatmap of upstream and downstream amino acids of all identified modification sites. Red indicates that this amino acid is significantly enriched near the modification site, and green indicates that this amino acid is significantly reduced near the modification site.

**Fig. S5** The protein levels of H4 and H3.3C in T2DM patients decreased compared to normal controls.

**Supplemental Table S1 Characteristics of 3 T2DM and 3 normal glucose tolerance (NGT) of proteomic analysis**

| Characteristics  Gender (M/F) | T2DM  (n=3) 1/2 | NGT  (n=3) 1/2 |
| --- | --- | --- |
| Age (years) | 57.3 ± 4.25 | 55.17 ± 5.10 |
| BMI (kg/m^2^) | 26.6 ± 3.11 | 24.3 ± 2.79 |
| FBG (mM) | 8.6± 2.15 | 4.3±1.07* |
| HbA1c (%) | 8.8± 1.06 | 5.4±0.92* |
| FINS (pM) | 68.54± 12.56 | 159.12± 30.18** |
| FCP (nM) | 0.36± 0.012 | 0.65± 0.019** |
| TG (mM) | 1.62± 1.04 | 1.14± 0.89 |
| CHO (mM) | 5.04± 2.12 | 3.72± 0.76 |
| HDL-C (mM) | 1.28± 0.54 | 1.57± 0.71 |
| LDL-C (mM) | 3.18± 1.86 | 2.91± 1.24 |
| HOMA-IR | 26.54± 2.74 | 34.68± 3.22* |

**Abbreviations:**  Data are mean ±standard deviation (SD).

T2DM, Type 2 diabetes mellitus; NGT, normal glucose tolerance; M, male; F, female; BMI, body mass index; FBG, fasting blood glucose; HbA1c, glycated hemoglobin; FINS, fasting insulin; FCP, fasting C-peptide; TG, triglycerides; CHO, cholesterol. LDL, low density lipoprotein; HDL, high density lipoprotein; HOMA-IR: insulin resistance index of the steady state model

**P*<0.05, ***P*<0.01 vs. NGT.

**Supplemental Table S2 Sequences of DNA primers**

| **Gene/GenBank** | **Protein** | **Primers** |
| --- | --- | --- |
| hApoB  NM_000384 | ApoB-100 | F:5’-GAG GTC ATC AGG AAG GGC TCA AAG-3’ |
|  |  | R:5’- GGG ATC ACC TCC GTT TTG GTG GTA-3’ |
| hHIST1H4A | Histone H4 | F:5’-AAGGGTTTGGGTAAGGGGG-3’ |
|  |  | F:5’-TAGATCAGACCAGAGATCCGC-3’ |
| hHistone H3.3C | Histone H3.3C | F:5’-GTAAGTAAGGAGGTCTCTGTACCATGGCT-3’ |
|  |  | F:5’-CCATCATAGTGGATTCTTAAGCACGTTCTC-3’ |
| hGAPDH | GAPDH | F:5’-TCTCTGCTCCTCCTGTTCGA-3’ |
|  |  | R: 5’- GCGCCCAATACGACCAAATC-3’ |

**Abbreviations:** ApoB, apolipoprotein B; HIST1H4A, Histone cluster 1; H4a. GAPDH, glyceraldehyde-3-phosphate dehydrogenase.

**Supplemental Table S3**  **Analysis using software**

| **Analysis** | **Software/Method** | **Version/URL** |
| --- | --- | --- |
| Mass spectrometry data analysis | MaxQuant | v.1.5.2.8 <http://www.maxquant.org/> |
| Motif analysis | [MoMo](http://meme-suite.org/tools/momo) | V5.0.2<http://meme-suite.org/tools/momo> |
| GO annotation | UniProt-GOA | http://www.ebi.ac.uk/GOA/ |
|  | [InterProScan](http://www.ebi.ac.uk/interpro/) | v.5.14-53.0 <http://www.ebi.ac.uk/interpro/> |
| Domain annotation | [InterProScan](http://www.ebi.ac.uk/interpro/) | v.5.14-53.0 <http://www.ebi.ac.uk/interpro/> |
| KEGG annotation | KAAS | v.2.0 <http://www.genome.jp/kaas-bin/kaas_main> |
|  | KEGG Mapper | V2.5 <http://www.kegg.jp/kegg/mapper.html> |
| Subcellular localization | [Wolfpsort](http://wolfpsort.seq.cbrc.jp/) | v.0.2 <http://www.genscript.com/psort/wolf_psort.html> |
|  | CELLO | v.2.5 <http://cello.life.nctu.edu.tw/> |
| Enrichment analysis | Perl module | v.1.31 https://metacpan.org/pod/Text::NSP::Measures::2D::Fisher |
| Cluster heat-map | R Package pheatmap | v.2.0.3 <https://cran.r-project.org/web/packages/cluster/> |
| Protein interaction | Blast | v.2.2.26 <http://blast.ncbi.nlm.nih.gov/Blast.cgi> |
|  | R package networkD3 | v.0.4 https://cran.r-project.org/web/packages/networkD3/ |

**Supplemental Table S4 Results of protein concentration**

| **Sample** | **Protein concentration**  **(μg / μL)** | **Volume**  **(μL)** | **Total protein**  **(μg)** |
| --- | --- | --- | --- |
| P6002 | 9.3 | 900 | 8379.0 |
| P6068 | 10.0 | 900 | 9003.4 |
| P6034 | 9.0 | 900 | 8144.9 |
| N6420 | 8.6 | 900 | 7774.1 |
| N6053 | 10.1 | 900 | 9081.5 |
| N6052 | 8.8 | 900 | 7944.9 |
